# The haemodynamic effects of crystalloid and colloid volume resuscitation on primary, derived and efficiency variables in post-CABG patients

**DOI:** 10.1186/s40635-019-0224-7

**Published:** 2019-03-04

**Authors:** S. Sondergaard, J. S. Larsson, P. W. Möller

**Affiliations:** 1 Centre of Elective Surgery, Department of Anaesthesia and Intensive Care, Silkeborg Regional Hospital, Silkeborg, Denmark; 2 Swedish Armed Forces, Stockholm, Sweden; 3 Department of Anaesthesiology and Intensive Care Medicine, Institute of Clinical Sciences at the Sahlgrenska Academy, University of Gothenburg, Sahlgrenska University Hospital Östra, Gothenburg, Sweden

**Keywords:** Haemodynamics, Methods, Physiology, Utilisation, Models, Cardiovascular, Fluid therapy

## Abstract

**Background:**

Recent studies in haemodynamic management have focused on fluid management and assessed its effects in terms of increase in cardiac output based on fluid challenges or variations in pulse pressure caused by cyclical positive pressure ventilation. The theoretical scope may be characterised as Starling-oriented. This approach ignores the actual events of right-sided excitation and left-sided response which is consistently described in a Guyton-oriented model of the cardiovascular system.

**Aim:**

Based on data from a previous study, we aim to elucidate the primary response to crystalloid and colloid fluids in terms of cardiac output, mean blood pressure and right atrial pressure as well as derived and efficiency variables defined in terms of Guyton venous return physiology.

**Method:**

Re-analyses of previously published data.

**Results:**

Cardiac output invariably increased on infusion of crystalloid and colloid solutions, whereas static and dynamic efficiency measures declined in spite of increasing pressure gradient for venous return.

**Discussion:**

We argue that primary as well as derived and efficiency measures should be reported and discussed when haemodynamic studies are reported involving fluid administrations.

## Background

Assessment of cardiovascular regulation in the perioperative period is a highly debated topic in anaesthesia concerning goals, evaluation and choice of therapy. The ultimate purpose of regulation is to ensure that oxygen delivery (DO_2_) does not rate-limit the oxygen consumption (VO_2_) by maintaining a cardiac output (CO) at prevailing haemoglobin (Hb) and oxygen saturation (S_a_O_2_). The attainment of a targeted CO may be accomplished using a number of strategies suggested by established algorithms based on an implicit Starling cardiocentric model. In this, the qualitative description of the cardiac function curve has three determinants: preload, afterload and contractility. *Volume resuscitation* is a means of increasing preload, ‘moving’ the volume responsive patient up the function curve in terms of preload vs CO. We observe the increase in CO with a cut-off at 10–15% (dictated by minimum discernible difference of CO monitor) in response to a volume increment of 250–500 mL of fluid. It is, however, often unspecified which type of fluid and whether the volume should be fixed, related to body size or adapted to the vascular compliance of the patient and which infusion rate should be used. *Inotropy* is another option increasing CO, and guidelines usually refer the clinician to the use of inotropic therapy when the preloading option does not confer a CO increase > 10%. In the Starling model, there is no option for quantifying the ‘cost’ of volume resuscitation, ɔ: how efficiently added volume results in an increase in cardiac power ((MAP − CVP) × CO), nor numerically characterise heart efficiency and changes therein.

Looking at cardiovascular regulation from a Guytonian, histocentric perspective (i.e. the oxygen demand of the tissues plays the major role in regulating venous return and consequently the cardiac output), the determinants of CO are volume (expressed as mean systemic filling pressure), vascular resistance and heart efficiency. These are well-defined and quantitative in the Parkin and Leaning haemodynamic model. When integrated into and presented bedside in a clinical decision support system (CDSS), they provide the option of quantifying the effects of and guide therapy.

This study used imported data from Skytte Larsson [1] and aims to describe similarities and differences in the course of crystalloid and colloid resuscitation. We analysed primary (MAP, CO, RAP), derived and efficiency variables to compare fluid resuscitation in coronary artery bypass patients seen from a Starling and Guytonian perspective.

## Patients and measurements

Thirty stable, post-operative CABG patients were allocated in random order after informed, signed consent to receive Ringer’s Acetate 20 mL/kg or Voluven 130/0.4 (Fresenius Kabi) 10 mL/kg over a period of 20–30 min to allow for stress relaxation. In a previous study, these volumes generated identical relative increases in CO and were regarded as equivalent in this sense. Prior to infusion, duplicate control baseline haemodynamic measurements were performed consisting of transcardiac thermodilution cardiac output (TDCO) in triplicate within 10%, mean arterial and central venous pressure (MAP, CVP) with pressure sensors aligned to phlebostatic axis at midaxillary level. MAP, CVP and CO are the *primary variables*. Arterial and venous blood gases were analysed. Post-infusion at 20, 40 and 60 min (*t*^20^, *t*^40^, *t*^60^) measurements were repeated. Patients were ventilated in volume-controlled mode with a PEEP of 5 cm H_2_O at a respiratory rate of 12–16 min^−1^ using a tidal volume 6–8 mL/kg to maintain normocarbia. Sedation was provided using propofol and morphine. Patient demographics are shown in Table 1. Haemodynamic and gas analysis data are available on request.

**Table 1:** Demographics, mean and standard deviation (SD) of patients

	Patients receiving	
	Crystalloid	Colloid
Gender (F/M)	3/12	1/14
Age, years	68 ± 12	66 ± 10
Length, cm	174 ± 8.5	176 ± 6.4
Weight, kg	78.7 ± 11	78 ± 8.6
BSA, m^2^	1.94 ± 0.18	1.94 ± 0.13

## Calculations

### Fluid retention

The retention of fluid (FR) is a complex function of fluid composition, osmotic pressure, intravascular pressure and disease state. The resultant ‘percentage fluid retained of transfused volume’ was estimated according to:

ΔBV = BV(Hb/Hb_t_) – BV; Hb, haemoglobin; BV, blood volume.

The amount of fluid retained in the blood is given by

FR (%) = 100 × ΔBV/infused volume.

BV was calculated according to the Nadler equation [2].

Men: BV = (0.3669 × H^3^) + (0.03219 × W) + 0.6041.

Women: BV = (0.3561 × H^3^) + (0.03308 × W) + 0.1833.

H, height, m; W, weight, kg.

### Derivation of Guytonian model variables

Central to the Guytonian model is the mean systemic filling pressure, *P*_ms_, originally introduced by Weber [3] and half a century later revived by Starling [4]. Guyton formalised the study of *P*_ms_ in a monumental series of investigations [5–9] summarised in his Cardiac Output and its Regulation. [10] Whereas Guyton defined *P*_ms_ as the equilibrated arteriovenous pressure at zero flow (fibrillation), a number of alternative methods have been suggested to elicit *P*_ms_ in the intact circulation. Some of these are interventional: the cardiovascular system is excited and its response is registered [11–17]. For validity, this carries the implication of the stability of all other variables entering the function of the cardiovascular system during the excitation. It is limited to sedated patients in fully controlled ventilation. *P*_ms_ has a normal value of 7–12 mmHg [9, 18].

The estimation of *P*_ms_ has been approached from a purely computational angle by Parkin and Leaning. [19, 20] Based on a simple circulatory model, the following equation is offered as an *analogue* to *P*_ms_: *P*_msa_ = 0.96 × CVP + 0.04 × MAP + *c* × CO, *c* is based on anthropometric data and attains values of 0.4–1.2. A normal value of *P*_msa_ is 7 mmHg as seen in a patient in whom CVP = 0, MAP = 100, CO = 6 and *c* = 0.5. *P*_msa_ = 0.96 × 0 + 0.04 × 100 + 0.5 × 6 = 7. [18]

**Fig. 1:**
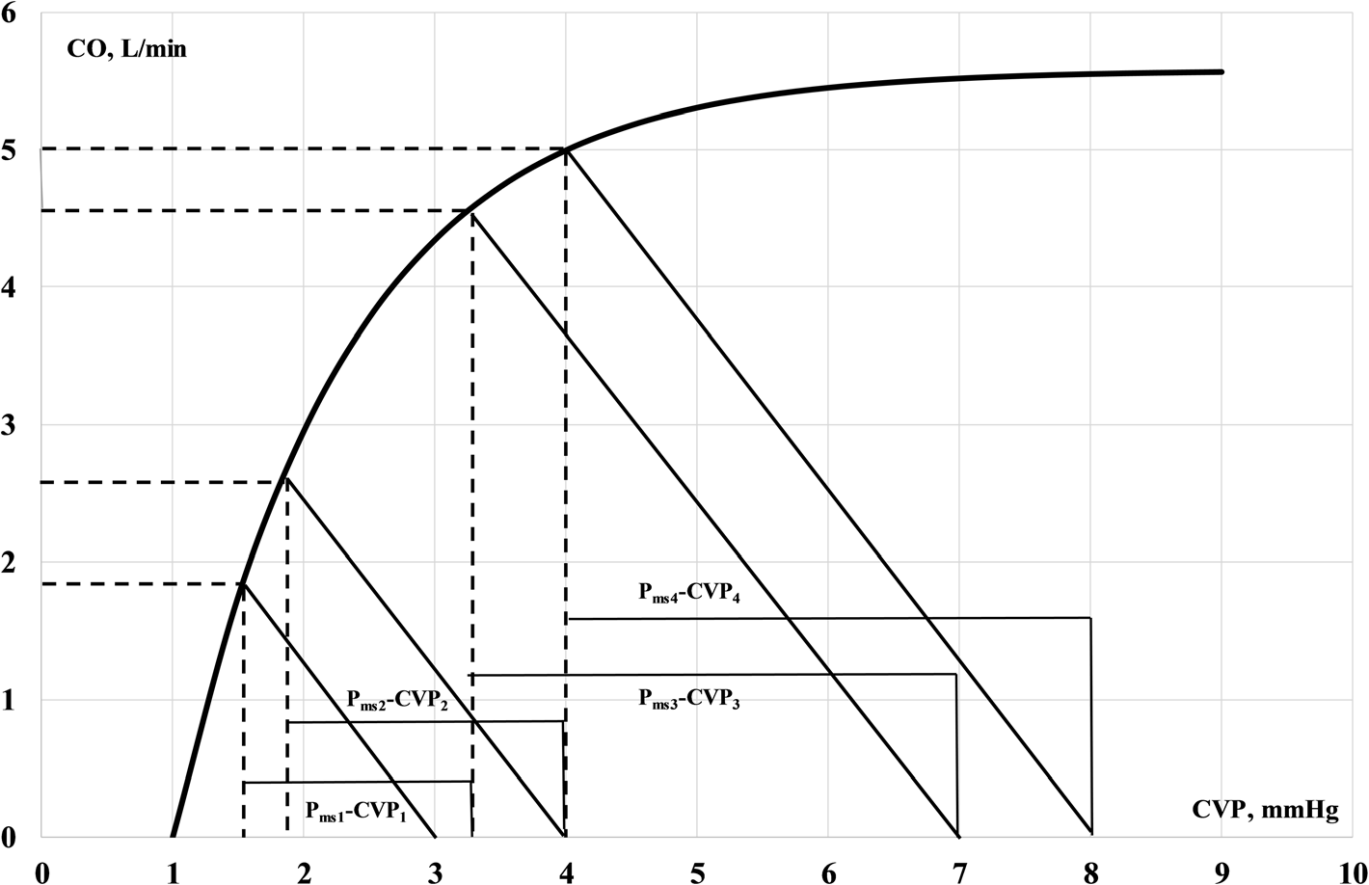
Two situations of venous return curves intersecting with cardiac function curve after fluid resuscitation. CO is on *y*-axis and CVP is on *x*-axis. In the first situation, marked with subscripts 1 and 2, a volume bolus increases *P*_msa_ from *P*_msa1_ to *P*_msa2_ and CVP from CVP_1_ to CVP_2_. The increase in return pressure (Δ (*P*_msa_ − CVP)) is large. Compare this favourable situation with the situation marked with subscripts 3 and 4: an identical increase in *P*_msa_ generates a larger increase in CVP and thus a smaller Δ (*P*_msa_ − CVP). Relating Δ (*P*_msa_ − CVP) to Δ*P*_msa_ (*E*_vol_) in the first instance yields a larger figure (app. 0.8) than in the second case (app. 0.25), indicating a better outcome of fluid resuscitation. This is formalised in Eq. (7). Heart efficiency, *E*_h_, declines from app. 0.5–0.6 in the first instance to 0.39–0.36 in the second

A fluid bolus invariably raises the mean systemic filling pressure (*P*_msa_) ceteris paribus. The outcome of fluid resuscitation is illustrated in Fig. 1 in the combination of Starling’s cardiac function curve and Guyton’s venous return curve. Depending on the position of the intercept of the venous return and the cardiac function curve, CVP and CO change in distinct patterns. If the venous return curves associated with Δ*P*_msa_ intercept with the ascending limb of the function curve, CVP rises minimally, while CO responds to the increase in the potential energy added to the venous capacitance. In contrast, if the venous return curves associated with Δ*P*_msa_ intercept the function curve in the flat part, the CO changes minimally and CVP increases substantially.

**Fig. 2:**
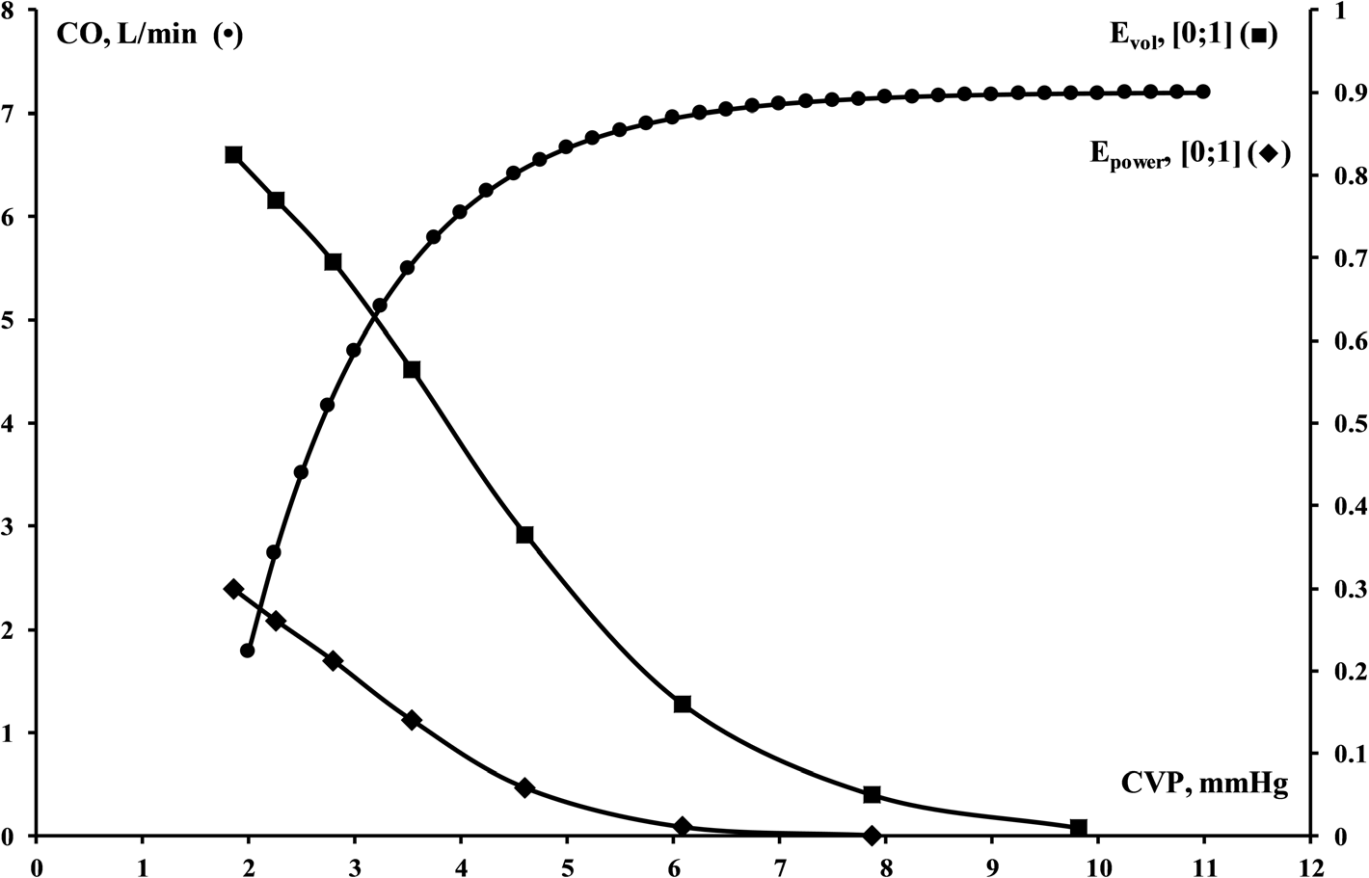
Concordant cardiac function curve (filled circle), volume efficiency (filled square) and power efficiency (filled diamond) as *P*_msa_ is increased stepwise by 2 mmHg. Power efficiency has been scaled by a factor 10 for visibility

In addition to *P*_msa_, the driving pressure for venous return (VRdP = *P*_msa_ − CVP) and power ((MAP−CVP) × CO) belong to derived variables. The assembly further allows for the definition of three efficiency variables, *E*_h_, *E*_vol_ and *E*_power_. *E*_h_, heart efficiency, expresses how well the heart handles the VRdP in terms of *P*_msa_ and CVP. Whereas *E*_h_ is a static variable, *E*_vol_ conveys a dynamic variable embodying the efficiency of added volume in terms of increase in VRdP related to increase in *P*_msa_. *P*_power_, power efficiency, dynamically describes the change in cardiac power in relation to the change in *P*_msa_. Notably, the variables are continuous in the interval [0;1] in contrast to the prevalent binary variable ‘volume responsiveness’: responder/non-responder. See Appendix for derivations of heart (*E*_h_), volume (*E*_vol_) and power efficiency (*E*_power_). The relationship between the evolution of CVP, *E*_vol_, *E*_power_ and the Starling cardiac function curve is illustrated in Fig. 2.

#### Statistical analysis

Primary, derived and efficiency variables were normally distributed and are characterised by mean and standard deviation; differences between time points and types of fluids were analysed by ANOVA with multiple comparisons and multiplicity adjusted *p* values, Tukey test, confidence intervals and significance. Analyses were performed using GraphPad Prism versions 6.05 and 8.01 (La Jolla, CA 92037, USA).

## Results

### Patients

### Fluid retention

**Fig. 3:**
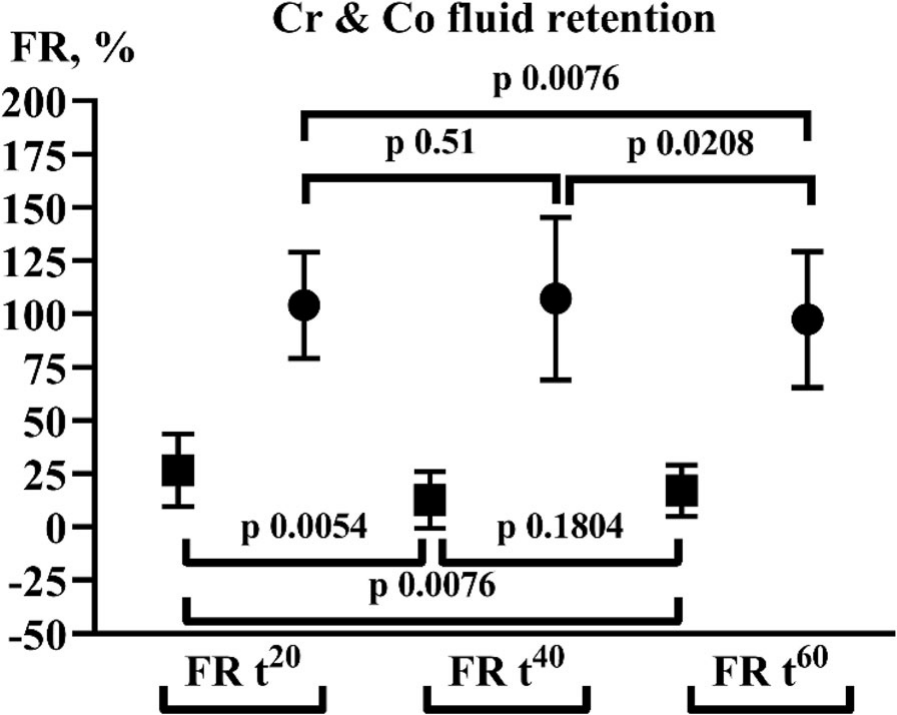
Fluid retention after one, two and three 20 min periods in the crystalloid (Cr, full square) and colloid (Co, full circle) group

Coefficients of variation in Hb were 13.7 and 13.6% in the crystalloid and 12.8 and 13.0% in the colloid baseline measurements. Percentages FR for colloid and crystalloid resuscitation are shown in Fig. 3. The differences between crystalloid FR at *t*^20^ to *t*^40^ and *t*^20^ to *t*^60^ were significant. The drop in colloid FR from *t*^40^ to *t*^60^ was significant. The differences between the fluid types were significant at all time points (*p* = < 0.0001). So, in short, crystalloid fluids leave the circulation, colloid stay.

### Primary variables

**Fig. 4:**
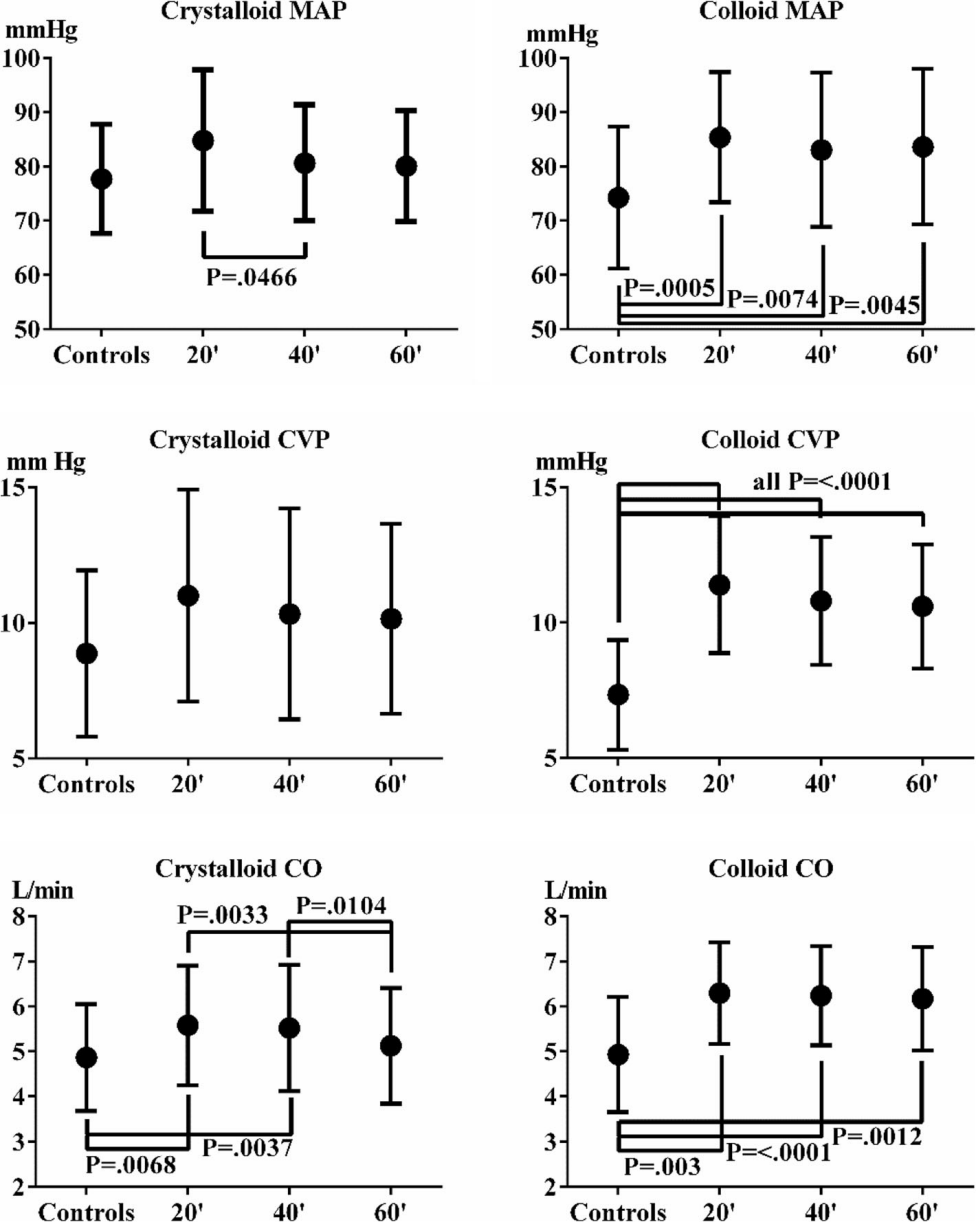
Temporal variation in primary variables MAP, CVP and CO during crystalloid and colloid resuscitation. Analysed as ANOVA with Tukey’s multiple comparisons. In all but one case (MAP), there were significant changes between time points in crystalloid MAP (decrease from *t*^20^ to *t*^40^) and CO (increase control to *t*^20^ and *t*^40^) and decrease *t*^20^ and *t*^40^ to *t*^60^). In the colloid series, increases from control to *t*^20^, *t*^40^ and *t*^60^ were seen in all three variables. The figures show mean ± SD

The primary variables MAP, CVP and CO increased significantly after colloid resuscitation. In the crystalloid group, this only happened in CO. This was followed by a significant decrease in CO in the crystalloid series. The average COs in crystalloid and colloid groups at baseline were 4.82 and 5.11 L/min. At *t*^20^, the mean COs were 5.58 and 6.30 L/min. Neither at baseline nor at *t*^20^, *t*^40^ and *t*^60^ were differences between crystalloid and colloid CO significantly different. The maximum ΔCO were 15.6 and 22% in the crystalloid and colloid groups. There were no other significant differences in MAP, RAP or CO at identical time points between crystalloid and colloid series, see Fig. 4.

### Derived variables

**Fig. 5:**
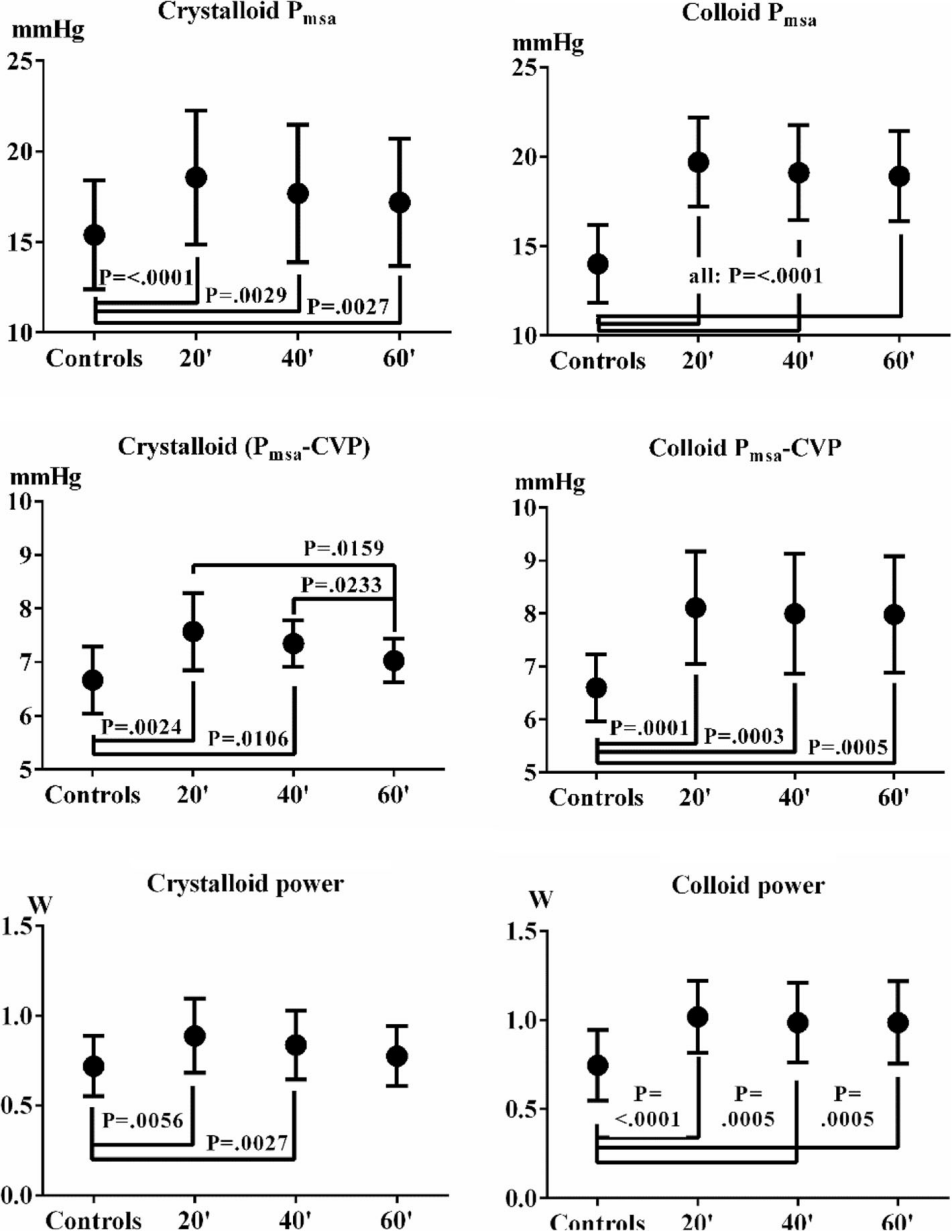
*P*_msa_, (*P*_msa_ − CVP) and power during crystalloid and colloid resuscitation. *P*_msa_ increased in both series and remained elevated for the time course of 60 min. The pressure gradient (*P*_msa_ − CVP) stayed elevated in the colloid series, but deteriorated from *t*^20^ onwards in the crystalloid series. Power increased significantly in both series but only stayed elevated in the colloid series

The *derived variables* *P*_msa_, and (*P*_msa_ − CVP) and power increased significantly. Power increased significantly in both series from control measurement to *t*^20^ and *t*^40^ in the crystalloid series after which power decreased. In the colloid series, power increases stayed significant for the duration of the experiment, see Fig. 5.

### Efficiency measures

**Fig. 6:**
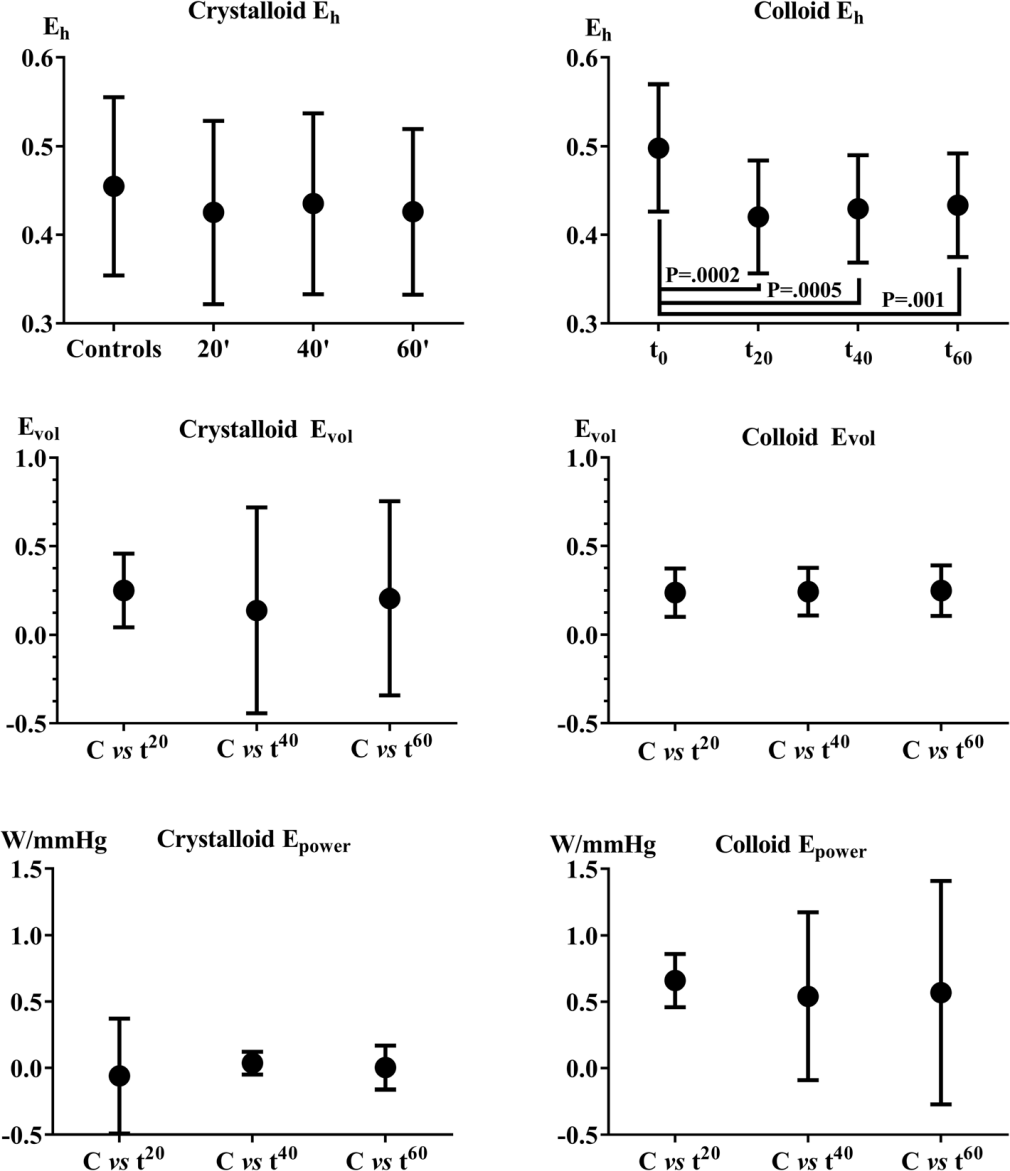
*E*_h_ did not change in the crystalloid series but decreased significantly in the colloid series indicating that the hearts were not able to convert the potential energy of *P*_msa_ into kinetic energy of MAP and CO but instead caused an increase in CVP, cp. Figure 2. Volume and power efficiency did not change significantly from control to timed stations. Power efficiency was close to zero in crystalloid series and approached ½ *W*/mmHg increase in *P*_msa_. The differences between crystalloid and colloid were significant in C vs *t*^20^ (*p* = 0.0003), C vs *t*^40^ (*p* = 0.0145) and C vs *t*^60^ (*p* = 0.005)

*E*_h_ as a static measure decreased significantly in the colloid group, but stayed constant in the crystalloid series. *E*_vol_, a dynamic measure, repeats the pattern of FR with significantly larger variance in crystalloid vs colloid group. *E*_vol_ did not demonstrate significant differences between time points in each series separately, nor differences between fluid types at identical time points. Insignificant declining efficiency is noted in crystalloid group, see Fig. 6.

**Fig. 7:**
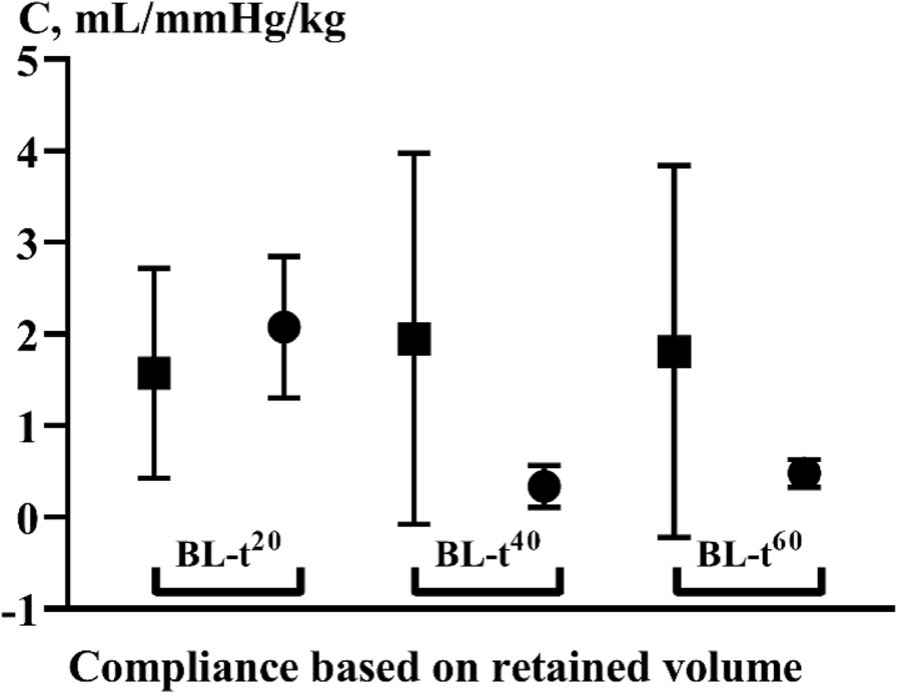
The compliances calculated from change in *P*_msa_ from baseline to *t*^20^, *t*^40^ and *t*^60^, and volume infused differed significantly between crystalloid and colloid (*p* = < 0.0001, 0.0013 and 0.0034) at identical time points. Crystalloid, full square; colloid, full circle

Results of compliance calculations are depicted in Figure 7.

## Discussion

The haemodynamic data originate from a study of the effect of resuscitation on renal perfusion and oxygenation. We aimed to illustrate the impact on primary, derived cardiovascular variables and efficiency measures. The purpose of the primary study was not to explore macrohaemodynamics by exciting the cardiovascular system. Our findings are co-incidental and not the result of any modification of goal-directed therapy (GDT). We will, however, discuss the results in the light of GDT as data collection is meticulous and detailed.

The analysis of cardiovascular effects of a crystalloid or colloid bolus in post-CABG patients demonstrated effects in primary variables (MAP, CO, CVP), derived variables (*C*, *P*_msa_, (*P*_msa_ − CVP), power) and the efficiency variables (*E*_h_, *E*_vol_, *E*_power_). The primary variables increased significantly from baseline through *t*^20^, *t*^40^ to *t*^60^ in the colloid series whereas only CO differed significantly through the crystalloid series—as CO deteriorated. The increase in CO is ascribed to the significant increase in *P*_msa_ and VRdP= (*P*_msa_ − CVP) in the colloid group.

Important differences were seen in fluid retention; these were reflected in the significant differences in compliance between the crystalloid and the colloid group. Notably, the crystalloid shows lower fluid retention and higher compliance. This, however, is understandable as the *P*_msa_ increases less while less fluid is retained. The calculation of FR is hampered by the low precision of determination of haemoglobin. This, however, affects crystalloid and colloid series in equal measure. The study used haemoglobin-derived FR in order to avoid the influence of changes in tonicity on mean corpuscular volume, MCV and haematocrit (Hct). An experiment with identical (incidentally) amounts of fluid changed plasma colloid oncotic pressure by 8.3% in voluven group and by − 26.2% in the crystalloid group. [20]. In effect, this should increase mean corpuscular volume, MCV, and Hct in crystalloid while decreasing MCV and Hct relatively in colloid resuscitation. Calculated compliance, normally 1–2 mL/kg/mmHg, therefore is a crude measure but concordant with previous results. [21] The low FR of crystalloid further means that the extravascular load of fluid may reach detrimental proportions in terms of tissue oedema resulting in reduced kidney, pulmonary, intestinal and hepatic function in addition to the immediate effect of certain crystalloids to degrade the glycocalyx (an effect they share with colloids!) and further promote extravasation. [22]

While the immediate effects on CO and MAP are the primary goals of fluid resuscitation, the scrutiny of the *efficiency measures* may answer the question whether fluid or other interventions will provide the haemodynamically most economical or appropriate course of action. This is a question hitherto only answered by the ‘maximisation concept’ in volume resuscitation based on the Starling cardiac function curve, and it is worthwhile to contemplate the meaning of ‘economical’ in this context. Studies from Starling [23] onwards relating the pressure-volume area (PVA) to oxygen consumption (VO_2_) arrive at a linear relationship in denervated conscious dogs. A higher preload in accordance with the Starling function curve leads to a higher CO and conceptually to a higher PVA and proportionally higher VO_2_. This linearity is valid at varied contractility (dobutamine infusion) in combination with variations in preload. [24] Thus, we cannot argue the ‘economy’ point from oxygen consumption.

Contemplate instead the aim of GDT: to guarantee oxygen delivery and adequate perfusion pressure for vital organs. The question of oxygen demand is rarely contemplated; rather it is supplanted with a dogma of optimisation/maximisation of flow. The dogma dictates that fluid is the sole means to optimise flow. A flow, which in the intact organism, is autoregulated according to VO_2_. Bundgaard-Nielsen [25, 26] fortuitously demonstrated this by volume loading awake, normal, unmedicated subjects with colloid. No increases in CO were observed using CardioQ. In another study, the author resuscitated anaesthetized patients with 200 mL colloid boli if SV increased > 10% from prior bolus. The volume needed was interpreted as ‘functional intravascular volume deficit’ and amounted to 200 to 600 mL. It is tempting to infer that in the first case, the intact organism redistributed the volume to the unstressed volume as the subjects were in no need of a higher CO or DO_2_. The splanchnic circulation has the ability to store added fluid as unstressed volume by increasing vascular capacitance dictated by the intact autoregulation of CO to VO_2_. In the second case, it is most probable that sympathetic reflexes and autoregulatory mechanisms have been attenuated by anaesthesia and that sympathicolysis has contributed to greater venous capacitance.

Circumstantial evidence can be drawn from passive leg raising (PLR). In the intact, unmedicated organism, PLR can be seen to increase CO in the short term (2–5 min). One textbook recommends that the assessment of volume responsiveness by PLR must be done within the first minute [27, 28] as ‘the maximal hemodynamic effects of PLR occur within the first minute of leg elevation.’ At this point, autoregulation kicks in and lowers CO, probably by the combined effect of redistributing volume to the unstressed compartment and lowering heart rate as a baroreceptor response [29]. The method of volume optimisation based on 10% increase in SV may even miss its own intention. The volume bolus must add pressure to the stressed volume and the pressure gradient for venous return in sufficient measure to affect an increase in CO of the stipulated 10% (ɔ: change the loading condition). It may further be hampered by the confounding response to haemodilution in terms of reflex increase in CO. [30, 31] The pressure addition is dictated by fluid retention, vascular compliance and the distribution of blood volume between systemic and splanchnic vascular beds. The increase in *P*_msa_ may be zero if added volume is deposited in increased venous capacitance.

‘Economy’ takes on a new meaning when addressing the efficiency variables. Heart efficiency, compliance, fluid state and resistance are never addressed by the adherents to the Starling-based maximisation concept. It has to be admitted that hitherto, there has been no discussion pertaining to these in Guytonian-oriented literature, there is absolutely no discussion as to branching values suggesting choice of intervention in terms of fluid, vaso- and/or cardioactive interventions. In the present analysis, evidently *E*_h_ significantly decreased as a result of the infused volume. *E*_h_ is a static measure, and there is no guideline as to which value should preclude the continued use of fluids and invite the use of inotropes or vasopressors. In the Navigator Clinical Decision Support System, an *E*_h_ < 0.3 is suggestive of the use of inotropes rather than fluids. In a clinical study relating (binary) volume responsiveness to (continuous) volume efficiency, the mean value of *E*_vol_ was 0.35 in responders and 0.1 in non-responders. [32] The ΔCO of 15–25% in the present study is obviously at the expense of a disproportionate increase in CVP signalling the increased distension of the right ventricle and the relative inability of the heart to eject the added volume. An increased CVP per se (ɔ: without a corresponding increase in *P*_ms_), furthermore, is associated with decreased drainage from venous beds, irrespective of arterial perfusion pressure. This creates a problem with increased bleeding during surgery severing venous plexuses. In the intensive care unit, an elevated CVP inhibits the drainage of kidney, intestines and lungs with known detrimental effects of kidney failure, impaired peristalsis and oedema. [33]

The dynamic counterpart to *E*_h_ is *E*_vol_. As in the case of *E*_h_, we have no safe guidance as to branching value in the decision pathway but a value in the interval 0.5 to 1 may be a suggestion.

We introduce the calculation of power efficiency, *E*_power_ showing the same pattern as *E*_h_ and *E*_vol_: slightly declining and with great variance in the crystalloid group and rather stable with low variance in the colloid group. *E*_power_ is derived from cardiovascular power as CO × MAP. This has been shown to correlate highly with mortality in the ICU. [34]

Intentionally, we have not made comparisons with prevailing methods of volume responsiveness (positive pressure ventilation induced changes in venous return reflected in left-sided variables as pulse pressure or stroke volume variation) as the physiological foundation of these is too inconsistent and garnered in caveats. [35, 36] The trend is toward abandoning them. [27]

## Conclusion

In conclusion, we have demonstrated the haemodynamic effects of crystalloid and colloid resuscitation in post-CABG patients. The primary variables MAP, RAP and CO beguile the clinician—in other circumstances—to assess that volume resuscitation was successful while the efficiency variables demonstrate that the added volume comes with the cost of actually straining the organism with a disproportionate increase in CVP. It is furthermore demonstrated that crystalloid volume has a highly variable and unpredictable effect dependent on its degree of fluid retention.

## Appendix

1 $$ \mathrm{Power}=\mathrm{CO}\times \left(\mathrm{MAP}-\mathrm{RAP}\right)\times 0.0022,W $$

*Efficiency variables*

*Volume efficiency*, *E*_vol_, may appropriately answer the question of whether fluid, vasopressor or inotropic therapy is more conducive to the target of increasing CO and by implication DO_2_.

2 $$ {E}_{\mathrm{vol}}=\frac{\Delta \left({P}_{\mathrm{msa}}-\mathrm{RAP}\right)}{\Delta {P}_{\mathrm{msa}}},\left[0;1\right] $$

Additionally, it may be of interest to characterise power efficiency, *E*_power_, in terms of the ratio between the change in power (MAP × CO) and the change in *P*_msa_.

3 $$ {E}_{\mathrm{power}}=\frac{\Delta \left(\left(\mathrm{MAP}-\mathrm{RAP}\right)\times \mathrm{CO}\right)\times 0.0022}{\Delta {P}_{\mathrm{msa}}},\mathrm{W}/\mathrm{mmHg},\left[0;1\right] $$

## Abbreviations

BV: Blood volume
C: Compliance
CABG: Coronary artery bypass grafting
CDSS: Clinical decision support system
CVP: Central venous pressure
DO_2_: Oxygen delivery
E_h_: Heart efficiency
E_power_: Power efficiency
E_vol_: Volume efficiency
FR: Fluid retention
GDT: Goal directed therapy
Hb: Haemoglobin
Hct: Haematocrit
ICU: Intensive care unit
MAP: Mean arterial pressure
PEEP: Positive end expiratory pressure
PLR: Passive leg raising
P_ms_: Mean systemic filling pressure
P_msa_: Mean systemic filling pressure analogue
PVA: Pressure-volume area
RAP: Right atrial pressure
S_a_O_2_: Arterial oxygen saturation
SV: Stroke volume
TDCO: Transcardiac thermodilution cardiac output
VO_2_: Oxygen consumption
